# Spatio‐temporal analysis of deaths from carbon monoxide poisoning in Iran between 2011 and 2018: An ecological study

**DOI:** 10.1002/hsr2.1785

**Published:** 2024-01-03

**Authors:** Yousef Alimohamadi, Danial Rahimi, Ahmad Mehri

**Affiliations:** ^1^ Health Research Center, Life Style Institute Baqiyatallah University of Medical Sciences Tehran Iran; ^2^ Department of Nursing, School of Nursing and Midwifery Mashhad University of Medical Sciences Mashhad Iran; ^3^ Department of Epidemiology, School of Public Health and Safety Shahid Beheshti University of Medical Science Tehran Iran

**Keywords:** carbon monoxide, CO poisoning, Iran, poisoning

## Abstract

**Background and Aims:**

Mortality caused by carbon monoxide every year threatens the lives of Iranian people, whose spatial and temporal distribution is not known for formulating prevention policies and interventions. This study was conducted to determine the trend of mortality rate changes due to carbon monoxide CO poisoning by Spatio‐temporal analysis in Iran from 2011 to 2018.

**Methods:**

An ecological study was conducted based on data from the reports at the National Center for Statistics of Iran for 8 years from 21 March 2011 to 21 March 2018. The number of deaths due to CO poisoning and the annual mortality rates of CO poisoning per 100,000 populations were calculated. To determine the geographical and temporal distribution of death caused by carbon monoxide, spatiotemporal statistical analysis was used.

**Results:**

A total of 6078 deaths were reported due to CO poisoning 4497 deaths were male (74%) and 1596 were female (26%) from 2011 to 2018. Both sexes' mortality rate due to CO poisoning was 1.26 from 2011 to 0.91 in 2018. According to the results, the overall male‐to‐female ratio was 2.8. The mortality rate due to CO Poisoning had a decreasing trend. However, this trend did not have a linear trend (*p* = 0.37). The highest mortality due to CO poisoning was seen in the northern and western provinces of Iran.

**Conclusion:**

Our results showed that the mortality rate due to CO poisoning had a decreasing trend during the understudied period. Also, most of the deaths due to CO poisoning occurred in the northern and western provinces of Iran. So, designing prevention programs as well as increasing people's awareness in these regions is recommended.

## 1 BACKGROUND

Poisoning is one of the major concerns of the health system in every country. Carbon monoxide poisoning is one of the kinds of poisoning that occurs when a person inhales too much carbon monoxide gas.^1^ This gas is produced by burning fuels such as wood, gasoline, propane, and natural gas.^2^, ^3^ When these fuels are burned in enclosed spaces without proper ventilation, carbon monoxide can build up to dangerous levels.^1^, ^4^ Symptoms of carbon monoxide poisoning include headache, dizziness, nausea, vomiting, weakness, confusion, and loss of consciousness. In severe cases, it can lead to seizures, coma, and even death.^5^ Treatment for carbon monoxide poisoning involves removing the person from the source of the gas and providing them with fresh air. In some cases, oxygen therapy may be needed to help the person breathe better. In severe cases, hyperbaric oxygen therapy may be necessary to increase the amount of oxygen in the blood and reduce the amount of carbon monoxide.^6^

In Iran, poisoning is one of the leading causes of hospitalization and death,^7^ and about 20% of hospital admissions are due to poisoning.^8^ Although there has been no study on the pattern of carbon monoxide poisoning in Iran, based on the latest study, the incidence of this poisoning in Iran is 38.91 per 100,000 people, with a case fatality rate of about 10%. The highest frequency of poisonings was in females and only 10% of those poisoned died.^9^ Carbon monoxide (CO) poisoning, as one of the lethal poisoning, is responsible for a large percentage of poisonings and accidental deaths.^10^

CO poisoning accounts for about 50,000 deaths annually in the US emergency department, with 38% of deaths due to CO poisoning between the ages of 10 and 19 years.^11^, ^12^ In Asia, CO poisoning is one of the leading causes of suicide.^13^ Some studies in Iran have also shown that about 10% of poisonings in Iran are due to CO and reported a mortality rate of 3.1 per 100,000 population in 2016.^14^ Another study showed that the mortality ratio of this poisoning was 11.6 per 1000 deaths in 2016.^9^ Iran, as one of the main producers of natural gas, has extensive use of natural gas domestically. Despite the widespread use of gas‐fired devices and their possible risks, especially the sudden death of CO poisoning in Iran, the mortality and the distribution of CO poisoning deaths in this country is still unknown and no study has investigated so far. To show the trends of CO poisoning deaths with the increased use of urban gas based in Iranian provinces over time, the purpose of this study was to determine the trend of mortality rate changes due to CO poisoning by Spatio‐temporal analysis in Iran from 2011 to 2018.

## 2 METHODS

### 2.1 Study area

Iran is a country in the Middle East with a total area of 1,648,195 km^2^. According to the last National Census in 2016, the total population of Iran is approximately 80 million. Iran has a common border with Armenia, Turkmenistan, and Azerbaijan in the North; Afghanistan and Pakistan in the East; the Persian Gulf and Gulf of Oman in the South; and Iraq and Turkey in the West.

### 2.2 Study design and used data set

An ecology analysis was conducted based on the data obtained from Iran's official reports on population. Deaths from CO poisoning data were obtained based on the reports of the Iranian Legal Medicine Organization (ILMO) for 8 years from March 21, 2011 to March 21, 2018. At the end of each year, the ILMO publishes deaths from CO reports on its website, by gender and province.^15^ Access to this data is free for all. Confirmation of CO deaths is based on autopsy evidence, examination of the bodies of victims by a specialist, and an emergency medical report after being transferred death cases to provincial ILMO. This method of confirmation in all provinces is based on similar guidelines, and there is no difference in how to diagnose the cause of death. These cases were classified based on the International Classification of Diseases, Tenth Revision (ICD‐10) code X47,^16^ and verified by ILMO. The number of deaths due to CO poisoning and the annual mortality rates of CO poisoning per 100,000 population were calculated. Calculation of rates per 100,000 inhabitants in Iran was performed using census data from 2011 to 2018.

### 2.3 Temporal trend analysis

To assess the trend of reported mortality rates the line plot of reported cases during the understudied period was used.

### 2.4 Spatial analysis

The unit of spatial analysis was different provinces of Iran. A Choropleth map was used to describe the distribution of mortality rate of CO poisoning cases (per 100,000 people) each year by the population of the province. This index is calculated for each province as this formula:

$$\text{The mortality rate in the year of}x:\frac{n\text{umber of new cases in year of}X}{\;\text{total population in year of}x}\times 100,000.$$

### 2.5 Hotspot identification

To identify clusters of fatal cases due to CO poisoning in different years, the Hotspot analysis was used. Hot spots present the clusters of under‐study events. This analysis was performed using the Getis‐Ord Gi statistics. A high score on this index combined with a lower *p* value indicates the clustering of understudy events. The Gi statistics formula is as follows:

$$G_{i}^{*}=\frac{\sum_{j=1}^{n}w_{i,j}x_{j}-\bar{X}\sum_{j=1}^{n}w_{i,j}}{S\sqrt{\frac{n\sum_{j=1}^{n}w_{i,j}^{2}-{\left(\sum_{j=1}^{n}w_{i,j}\right)}^{2}}{n-1}}},$$

where *Xj* is the mortality rate of understudy events for province *j*, *w i.j* is the spatial weight between provinces *i* and *j*, and *n* is the total number of provinces. *X* and *S*, are calculated as follows:

$$\bar{X}=\frac{\sum_{j=1}^{n}x_{j}}{n},$$

$$S=\sqrt{\frac{\sum_{j=1}^{n}x_{j}^{2}}{n}-{\left(\bar{X}\right)}^{2}}.$$

In terms of hot spot analysis, the *α*: 0.01 and 0.05 were considered significant levels. All analyses were performed using ArcGIS version 10.5 and Excel version 2010.

## 3 RESULTS

### 3.1 Descriptive analysis

**Figure 1:**
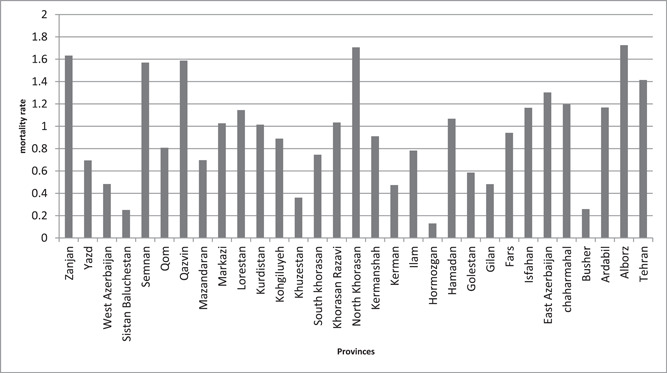
The average mortality rate of CO poisoning among provinces of Iran from 2011 to 2018 (per 100,000).

As shown in Table 1, A total of 6078 deaths were reported due to CO poisoning 4497 deaths were male (74%) and 1596 were female (26%) from 2011 to 2018. In both sexes, the mortality rate of CO poisoning was 1.26 from 2011 to 0.91 in 2018. According to the results, the overall male‐to‐female ratio was 2.8. As Figure 1 demonstrated, the average mortality rate due to CO poisoning in Semnan, North Khorasan, Qazvin, Zanjan, Alborz, and Tehran was higher than in other provinces of Iran.

**Table 1:** Number of death and mortality rates due to CO poisoning by gender in Iran between 2011 and 2018.

Christian year	Persian year	Population (million)			Death (*n*)			Mortality rate per 100,000 population			
		Female	Male	Both sex	Male	Female	Both sexes	Male	Female	Both sexes	Male/female
2011	1390	37,244	37,906	75,150	712	253	950	1.88	0.68	1.26	2.76
2012	1391	37,672	38,403	76,075	490	207	697	1.28	0.55	0.92	2.33
2013	1392	38,101	38,915	77,016	588	240	828	1.51	0.63	1.08	2.40
2014	1393	38,536	39,434	77,970	468	158	626	1.19	0.41	0.80	2.90
2015	1394	38,979	39,961	78,940	482	144	626	1.21	0.37	0.79	3.27
2016	1395	39,428	40,498	79,926	620	216	836	1.53	0.55	1.05	2.78
2017	1396	40,021	41,049	81,070	564	202	766	1.37	0.50	0.94	2.74
2018	1397	40,546	41,538	82,084	573	176	749	1.38	0.43	0.91	3.21

### 3.2 Temporal analysis

**Figure 2:**
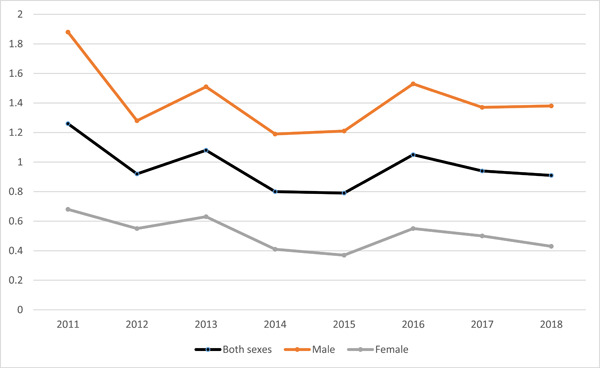
The time trend of mortality rate due to CO Poisoning from 2011 to 2018 in Iran by gender.

As Figure 2, in both sexes, the time trend in mortality rate due to CO Poisoning had fluctuation over time. The lowest and highest mortality rate was reported in 2014 and 2011, respectively, in both sexes. In addition to this visual inspection analysis, the mortality rate due to CO Poisoning had a decreasing trend during the understudied period.

### 3.3 Spatial distribution

**Figure 3:**
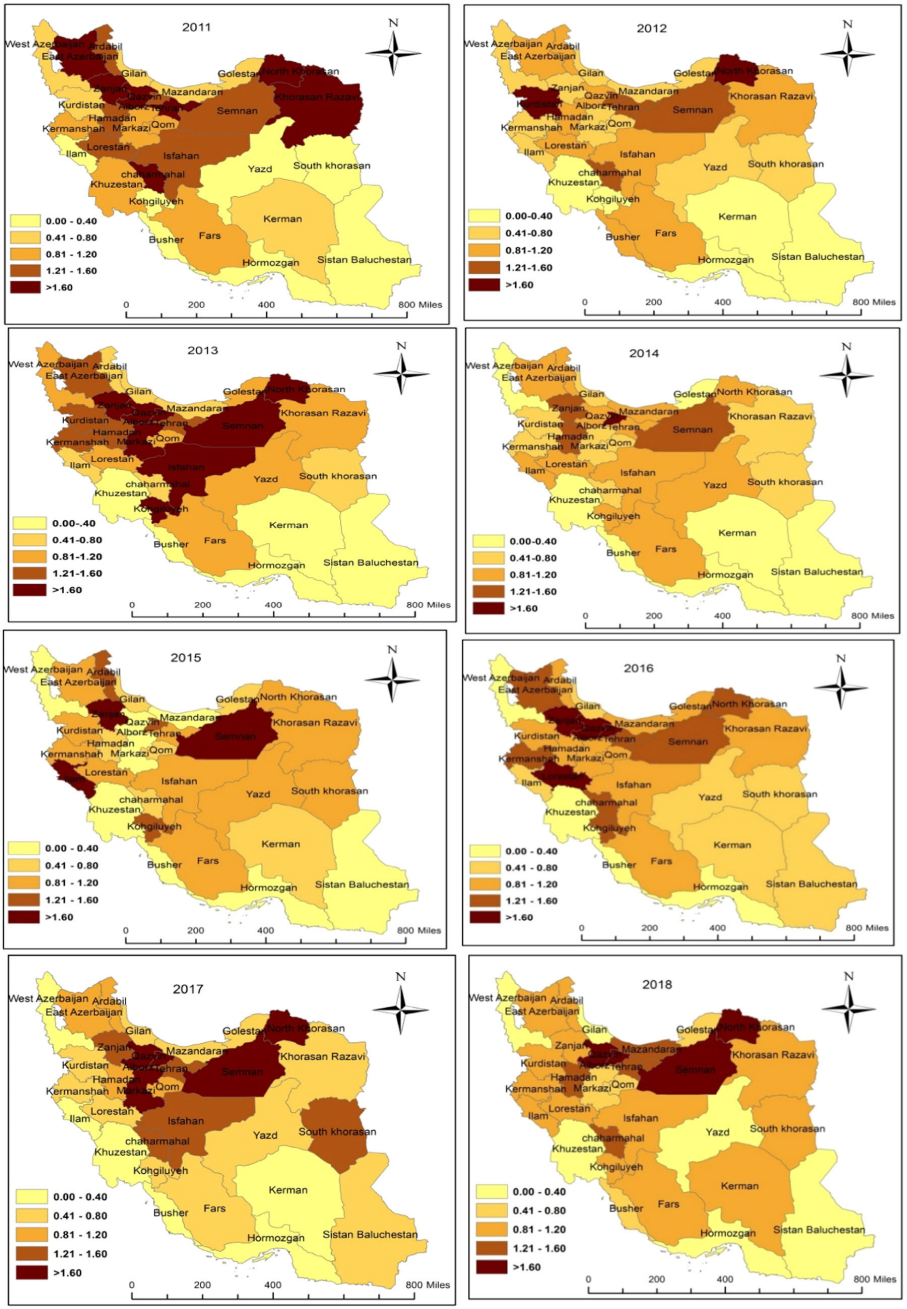
The mortality rate due to CO Poisoning among Iranian population from 2011 to 2018 by provinces (per 100,000).

Figure 3 shows that some provinces have had higher mortality rates than others in different years. Among all provinces of Iran, North Khorasan, Semnan, Qom, Qazvin, Zanjan, Chaharmahal Bakhtiari, and Tehran had the highest mortality rates between 2011 and 2018. The lowest mortality rates are also found in southern provinces of Iran, such as Kerman, Sistan and Baluchestan, Hormozgan, Bushehr, and Khuzestan.

### 3.4 Hot spots

**Figure 4:**
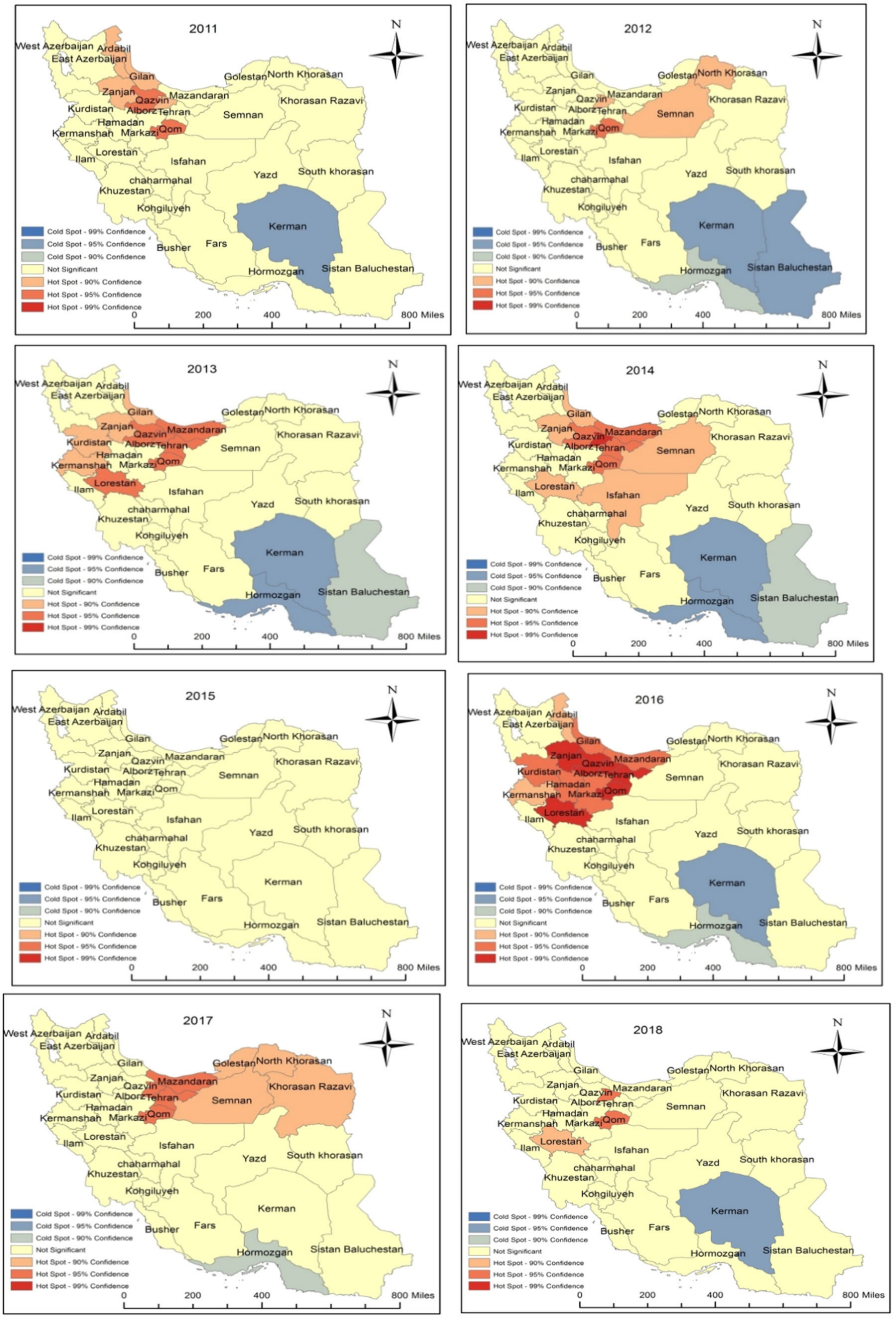
The Identified Hot Spot for mortality rate due to CO Poisoning among Iranian population from 2011 to 2018 by provinces (per 100,000).

In 2011, Qazvin and Qom were considered as hot spots. It means that the mortality rate due to CO poisoning (per 100,000) in these provinces was significantly higher than the overall average of the country, so these provinces were considered as hot spots (*p* < 0.05). In 2012, the Qom was considered as hot spot area (*p* < 0.05). Mazandaran, Qazvin, Tehran, Qom, and Lorestan provinces were considered as Hot spot in 2013(*p* < 0.05). Among provinces, Alborz had the most mortality rate in comparison to the country average, so was considered as a hot spot (*p* < 0.01) in 2014. Although there were not any Hot spot areas in 2015, there were hot spots in Qazvin, Tehran, Qom, and Lorestan provinces in 2013 (*p* < 0.01). Also, among provinces, Mazandaran, Tehran, and Qom were considered Hot spots in 2017 (*p* < 0.05), and finally, in 2018, Alborz and Qom were considered as hot spots (*p* < 0.05) (Figure 4).

## 4 DISCUSSION

CO poisoning is considered one of the health emergencies for its lethal nature as well as its subsequent complications if survived. This study aimed to investigate the trend of deaths due to CO poisoning among provinces of Iran. The results showed that the mortality rate due to CO poisoning was from 1.26 in 2011 to 0.91 per 100,000 populations in 2018, and the mortality trend did not change in these 8 years. Although there are no studies in the same period to compare these findings with others, some studies indicate that the trend of changes has not been significant before 2011. In a study carried out in Iran in 2010,^17^ the mortality rate due to CO poisoning was between 1.1 and 2.2 per 100,000, which was not statistically significant between 2002 and 2006. In another study by Nazari et al.,^18^ the mortality rate due to CO poisoning was less than 1 per 1000 people, with no statistical difference between 2003 and 2008. Since the ILMO approved the data in this study and there is no underreporting in the data collection, it seems that overall, there has not been little change in the mortality rate of CO poisoning in Iran over the years. However, the mortality rate of CO poisoning in Iran may have not increased. The mortality rate is still greater than in many other nations, though. This suggests that more needs to be done to protect Iranians from CO poisoning.

Our results demonstrated both the number of deaths and the mortality rate due to CO poisoning were higher in men than women each year. Consistent with our study, studies of Mirahmadizadeh et al.,^19^ Nazari et al.,^18^ and Shokrzadeh et al.^20^ have shown mortality rate due to CO poisoning was higher in men than women. Previous studies in other countries have also indicated these differences.^21^, ^22^, ^23^, ^24^ Although these differences are unclear, it seems men have more risky activities, such as working indoors or in garages with combustible tools than women which increases the exposure duration.^25^, ^26^ Studies to compare occupational among men and women who died, the mechanism of the effect of CO on Dying, and its differences between women and men, may help to understand this difference better.

This study showed that most of the deaths due to CO poisoning are higher in the northern and western provinces of Iran. As the northern and western provinces of Iran have a colder climate and many fatality cases accrued in the cold season,^14^, ^27^, ^28^ the use of gas heaters in these areas is higher than elsewhere, which could increase the exposure of the population to CO. To confirm this, a study conducted in the northern and western parts of Iran has shown that most deaths are due to CO poisoning and have been in cold seasons.^18^, ^29^ On the other hand, increasing the number of villages and cities with urban gas distribution networks in the north and west of Iran can also increase the number of poisoning cases and deaths due to CO. lack of understanding in using the gas and how to properly maintain appliances may contribute to risk.^18^ In support of this, our study showed that the mortality rate due to CO poisoning is lower in southern provinces of the country with warmer climates and provinces with less gas distribution network than in other regions of Iran.

### 4.1 Study limitations

There were some limitations to our study. First, this study is based on data annually published by ILMO by province and gender. The classification of this data is not based on other variables. We did not have additional data on seasonal distribution, occupational distribution, demographic characteristics, and the number of poisoned cases, which could better explain the epidemiological characteristics. Second, there were no adequate studies using data other than ILMO to compare these findings with the theme. Although the ILMO records information on carbon monoxide poisoning deaths, some incidents may have gone unreported. Additionally, since the information from the ILMO is self‐reported, there may be mistakes.

## 5 CONCLUSIONS

This study is one of the first studies to compare the spatial and temporal mortality rates due to CO poisoning in Iran. As our findings showed, mortality changes over time were not significant, and the mortality rate was higher in the northern and western provinces than in the south of Iran. Paying attention to general education about the principles of safety in the installation of heaters and the use of the gas networks, continuous and accurate monitoring of the installation and operation of CO‐producing, and the use of sensitive alarms can reduce mortality and morbidity due to CO. The results imply that Iran needs to increase awareness of CO poisoning and its prevention. The government could also enact regulations to increase the security of gas‐fired appliances. To provide a broader context for interpretation, it could be further developed to compare the study results with the body of knowledge of CO poisoning in Iran and other nations. This study suggests for future studies to determine the factors affecting the difference in deaths caused by CO poisoning. Also, in the next studies, it is suggested to investigate the impact of public safety policies in reducing the incidence of death due to CO poisoning.

## AUTHOR CONTRIBUTIONS

**Yousef Alimohamadi**: Conceptualization; formal analysis; investigation; methodology; writing—original draft. **Danial Rahimi**: Data curation; writing—original draft. **Ahmad Mehri**: Conceptualization; data curation; formal analysis; methodology; project administration; supervision; writing—review and editing.

## CONFLICT OF INTEREST STATEMENT

The authors declare no conflict of interest.

## TRANSPARENCY STATEMENT

The lead author Ahmad Mehri affirms that this manuscript is an honest, accurate, and transparent account of the study being reported; that no important aspects of the study have been omitted; and that any discrepancies from the study as planned (and, if relevant, registered) have been explained. All authors have read and approved the final version of the manuscript [CORRESPONDING AUTHOR or MANUSCRIPT GUARANTOR] had full access to all of the data in this study and take complete responsibility for the integrity of the data and the accuracy of the data analysis.

## Data Availability

The data that support the findings of this study are available from the corresponding author upon a reasonable request. Data derived from public domain resources.
